# Cleaning and Disinfectant Chemical Exposures and Temporal Associations with COVID-19 — National Poison Data System, United States, January 1, 2020–March 31, 2020

**DOI:** 10.15585/mmwr.mm6916e1

**Published:** 2020-04-24

**Authors:** Arthur Chang, Amy H. Schnall, Royal Law, Alvin C. Bronstein, Jeanna M. Marraffa, Henry A. Spiller, Hannah L. Hays, Alexandra R. Funk, Maria Mercurio-Zappala, Diane P. Calello, Alfred Aleguas, Douglas J. Borys, Tegan Boehmer, Erik Svendsen

**Affiliations:** ^1^Division of Environmental Health Science and Practice, National Center for Environmental Health, CDC; ^2^Division of Analysis, Research, and Practice, National Center for Injury Prevention and Control, CDC; ^3^Department of EMS and Injury Prevention, Hawaii Department of Health; ^4^Department of Emergency Medicine, Upstate Medical University, Upstate New York Poison Center, Syracuse, New York; ^5^Central Ohio Poison Center, Nationwide Children’s Hospital, Columbus, Ohio; ^6^New York City Poison Control Center, New York; ^7^New Jersey Poison Information and Education System, Rutgers New Jersey Medical School, Newark, New Jersey; ^8^Florida Poison Information Center – Tampa, Florida; ^9^School of Pharmacy, Concordia University Wisconsin, Mequon, Wisconsin; Wisconsin Poison Center, Milwaukee, Wisconsin.

On January 19, 2020, the state of Washington reported the first U.S. laboratory-confirmed case of coronavirus disease 2019 (COVID-19) caused by infection with SARS-CoV-2 (*1*). As of April 19, a total of 720,630 COVID-19 cases and 37,202 associated deaths* had been reported to CDC from all 50 states, the District of Columbia, and four U.S. territories (*2*). CDC recommends, with precautions, the proper cleaning and disinfection of high-touch surfaces to help mitigate the transmission of SARS-CoV-2 (*3*). To assess whether there might be a possible association between COVID-19 cleaning recommendations from public health agencies and the media and the number of chemical exposures reported to the National Poison Data System (NPDS), CDC and the American Association of Poison Control Centers surveillance team compared the number of exposures reported for the period January–March 2020 with the number of reports during the same 3-month period in 2018 and 2019. Fifty-five poison centers in the United States provide free, 24-hour professional advice and medical management information regarding exposures to poisons, chemicals, drugs, and medications. Call data from poison centers are uploaded in near real-time to NPDS. During January–March 2020, poison centers received 45,550 exposure calls related to cleaners (28,158) and disinfectants (17,392), representing overall increases of 20.4% and 16.4% from January–March 2019 (37,822) and January–March 2018 (39,122), respectively. Although NPDS data do not provide information showing a definite link between exposures and COVID-19 cleaning efforts, there appears to be a clear temporal association with increased use of these products.

The daily number of calls to poison centers increased sharply at the beginning of March 2020 for exposures to both cleaners and disinfectants (Figure). The increase in total calls was seen across all age groups; however, exposures among children aged ≤5 years consistently represented a large percentage of total calls in the 3-month study period for each year (range = 39.9%–47.3%) (Table). Further analysis of the increase in calls from 2019 to 2020 (3,137 for cleaners, 4,591 for disinfectants), showed that among all cleaner categories, bleaches accounted for the largest percentage of the increase (1,949; 62.1%), whereas nonalcohol disinfectants (1,684; 36.7%) and hand sanitizers (1,684; 36.7%) accounted for the largest percentages of the increase among disinfectant categories. Inhalation represented the largest percentage increase from 2019 to 2020 among all exposure routes, with an increase of 35.3% (from 4,713 to 6,379) for all cleaners and an increase of 108.8% (from 569 to 1,188) for all disinfectants. Two illustrative case vignettes are presented to highlight the types of chemical exposure calls managed by poison centers.

**FIGURE:**
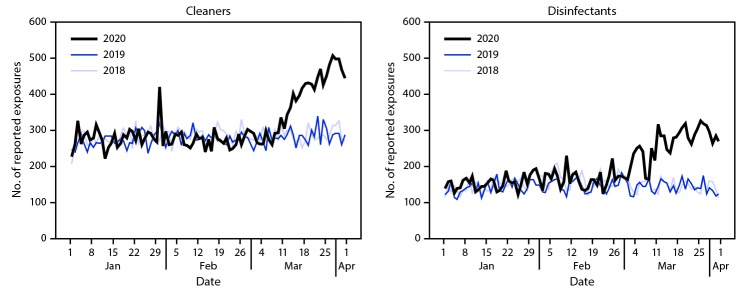
Number of daily exposures to cleaners and disinfectants reported to U.S. poison centers — United States, January–March 2018, 2019, and 2020*^,†^ * Excluding February 29, 2020. ^†^ Increase in exposures to cleaners on January 29, 2020, came from an unintentional exposure to a cleaning agent within a school.

**TABLE:** Number and percentage of exposures to cleaners and disinfectants reported to U.S. poison centers, by selected characteristics — United States, January–March 2018, 2019, and 2020

Characteristic	No. (%)					
	Cleaners			Disinfectants		
	2018	2019	2020	2018	2019	2020
**Total**	**25,583 (100.0)**	**25,021 (100.0)**	**28,158 (100.0)**	**13,539 (100.0)**	**12,801 (100.0)**	**17,392 (100.0)**
**Age group (yrs)**						
0–5	10,926 (42.7)	10,207 (40.8)	10,039 (35.7)	7,588 (56.0)	6,802 (53.1)	8,158 (46.9)
6–19	2,655 (10.4)	2,464 (9.8)	2,516 (8.9)	1,803 (13.3)	1,694 (13.2)	2,358 (13.6)
20–59	8,072 (31.6)	8,203 (32.8)	9,970 (35.4)	2,659 (19.6)	2,791 (21.8)	4,056 (23.3)
≥60	1,848 (7.2)	1,936 (7.7)	2,356 (8.4)	929 (6.9)	848 (6.6)	1,455 (8.4)
Unknown	2,082 (8.1)	2,211 (8.8)	3,277 (11.6)	560 (4.1)	666 (5.2)	1,365 (7.8)
Exposure route*						
Ingestion	16,384 (64.0)	15,710 (62.8)	16,535 (58.7)	11,714 (86.5)	10,797 (84.3)	13,993 (80.5)
Inhalation	4,747 (18.6)	4,713 (18.8)	6,379 (22.7)	540 (4.0)	569 (4.4)	1,188 (6.8)
Dermal	4,349 (17.0)	4,271 (17.1)	4,785 (17.0)	1,085 (8.0)	1,078 (8.4)	1,695 (9.7)
Ocular	3,355 (13.1)	3,407 (13.6)	3,802 (13.5)	984 (7.3)	1,067 (8.3)	1,533 (8.8)
Other/Unknown	182 (0.7)	169 (0.7)	166 (0.6)	89 (0.7)	95 (0.7)	147 (0.8)

*Exposure might have more than one route.

## Case 1

An adult woman heard on the news to clean all recently purchased groceries before consuming them. She filled a sink with a mixture of 10% bleach solution, vinegar, and hot water, and soaked her produce. While cleaning her other groceries, she noted a noxious smell described as “chlorine” in her kitchen. She developed difficulty breathing, coughing, and wheezing, and called 911. She was transported to the emergency department (ED) via ambulance and was noted to have mild hypoxemia and end-expiratory wheezing. She improved with oxygen and bronchodilators. Her chest radiograph was unremarkable, and she was discharged after a few hours of observation.

## Case 2

A preschool-aged child was found unresponsive at home and transported to the ED via ambulance. A 64-ounce bottle of ethanol-based hand sanitizer was found open on the kitchen table. According to her family, she became dizzy after ingesting an unknown amount, fell and hit her head. She vomited while being transported to the ED, where she was poorly responsive. Her blood alcohol level was elevated at 273 mg/dL (most state laws define a limit of 80 mg/dL for driving under the influence); neuroimaging did not indicate traumatic injuries. She was admitted to the pediatric intensive care unit overnight, had improved mental status, and was discharged home after 48 hours.

The findings in this report are subject to at least two limitations. First, NPDS data likely underestimate the total incidence and severity of poisonings, because they are limited to persons calling poison centers for assistance. Second, data on the direct attribution of these exposures to efforts to prevent or treat COVID-19 are not available in NPDS. Although a causal association cannot be demonstrated, the timing of these reported exposures corresponded to increased media coverage of the COVID-19 pandemic, reports of consumer shortages of cleaning and disinfection products (*4*), and the beginning of some local and state stay-at-home orders.

Exposures to cleaners and disinfectants reported to NPDS increased substantially in early March 2020. Associated with increased use of cleaners and disinfectants is the possibility of improper use, such as using more than directed on the label, mixing multiple chemical products together, not wearing protective gear, and applying in poorly ventilated areas. To reduce improper use and prevent unnecessary chemical exposures, users should always read and follow directions on the label, only use water at room temperature for dilution (unless stated otherwise on the label), avoid mixing chemical products, wear eye and skin protection, ensure adequate ventilation, and store chemicals out of the reach of children.

## Footnotes

* Total cases include 1,282 probable cases, and total deaths include 4,226 probable associated deaths.

## References

[1] Holshue ML, DeBolt C, Lindquist S, ; Washington State 2019-nCoV Case Investigation Team. First case of 2019 novel coronavirus in the United States. N Engl J Med 2020;382:929–36. 32004427 10.1056/NEJMoa2001191 PMC7092802

[2] CDC. Coronavirus disease 2019 (COVID-19): cases in US. Atlanta, GA: US Department of Health and Human Services, CDC; 2020. https://www.cdc.gov/coronavirus/2019-ncov/cases-updates/cases-in-us.html

[3] CDC. Coronavirus disease 2019 (COVID-19): cleaning and disinfecting your home. Atlanta, GA: US Department of Health and Human Services, CDC; 2020. https://www.cdc.gov/coronavirus/2019-ncov/prevent-getting-sick/disinfecting-your-home.html

[4] Guynn J. Looking for Lysol spray and Clorox wipes? COVID-19 wiped out disinfectants, but here’s when you can buy again. McLean, VA: USA Today; 2020. https://www.usatoday.com/story/money/2020/04/09/coronavirus-clorox-lysol-shortages-walmart-costco-publix-winco-lowes/2961818001/

